# Dietary Patterns and Feeding Behavior of Infants in Croatia: Findings from the National Food Consumption Survey on Infants and Children

**DOI:** 10.3390/children12091125

**Published:** 2025-08-26

**Authors:** Ana Ilić, Ivana Rumbak, Martina Pavlić, Nataša Šarlija, Lidija Šoher, Daniela Čačić Kenjerić, Jasna Pucarin-Cvetković, Darja Sokolić

**Affiliations:** 1 Department of Food Quality Control, University of Zagreb Faculty of Food Technology and Biotechnology, University of Zagreb, Pierottijeva Ulica 6, 10000 Zagreb, Croatia; ana.ilic@pbf.unizg.hr; 2 Centre for Food Safety, Croatian Agency for Agriculture and Food, Ivana Gundulića 36b, 31000 Osijek, Croatia; martina.pavlic@hapih.hr (M.P.); darja.sokolic@hapih.hr (D.S.); 3 Department of Management, Organization and Entrepreneurship, Faculty of Economics in Osijek, Josip Juraj Strossmayer University of Osijek, Trg Ljudevita Gaja 7, 31000 Osijek, Croatia; natasa.sarlija@efos.hr; 4 Department of Food and Nutrition Research, Faculty of Food Technology Osijek, Josip Juraj Strossmayer University of Osijek, Franje Kuhača 18, 31000 Osijek, Croatia; lidija.soher@ptfos.hr (L.Š.); daniela.kenjeric@ptfos.hr (D.Č.K.); 5 Department of Environmental and Occupational Health and Sports Medicine, Andrija Štampar School of Public Health, School of Medicine University of Zagreb, Rockefeller St. 4, 10000 Zagreb, Croatia; jasna.pucarin@snz.hr; 6 Division for Environmental Health, Croatian Institute of Public Health, Rockefeller St. 7, 10000 Zagreb, Croatia

**Keywords:** breastfeeding, complementary feeding, Croatia, dietary intake, dietary patterns, infants, national survey

## Abstract

**Background/Objectives**: To prevent nutritional depletion and impaired weight status in infants, targeted public health policies and prevention programs based on scientific evidence are needed. This study provides an overview of the dietary patterns and feeding behavior of infants in Croatia as part of the National Food Consumption Survey on Infants and Children. **Methods:** This cross-sectional study was conducted following the EU Menu methodology and included 322 healthy infants (54% boys; aged 3 months up to 12 months) from Croatia. Two-day dietary records were collected and analyzed using NutriCro 2.0 software. **Results:** The daily energy intake of infants was on average 886 ± 219 kcal, mainly from carbohydrates (47.0%), followed by fat (41.6%) and protein (9.9%). The main sources of energy and macronutrients were milk and dairy products, grains, grain products, potatoes and tubers and the fruit food group. One third of infants were breastfed, and more than 70% of infants were introduced to complementary foods. Parents started complementary feeding at the age of 5.37 ± 0.82 months, mostly with vegetables. Breastfeeding was associated with higher energy intake, especially in infants younger than 6 months, while formula feeding was associated with lower energy intake. The multivariate regression models showed age-related interactions that attenuated the patterns for energy and macronutrient intake. **Conclusions:** The study emphasizes that milk and dairy products are the main source of energy and macronutrients. The study highlights the important role of breastfeeding in promoting higher energy intake in early infancy and the decreasing effect of infant formula consumption with age. These results can be used as a basis for health policies, programs and strategies that address infant feeding habits in Croatia.

## 1. Introduction

In the first year of life, infants have a high growth velocity, and the energy they need for adequate growth can account for 35% of daily energy requirements in the first three months, 17.5% in the fourth to sixth months of life and about a third by twelve months of age [1]. To meet the energy needs of infants and prevent malnutrition or obesity, the World Health Organization (WHO) recommends that infants be exclusively breastfed for the first six months. Thereafter, from the age of 6 months, the gradual introduction of complementary foods is recommended, while breastfeeding should be continued until the age of 2 years [2]. The timing of the introduction of complementary foods depends on the parents’ decision, which should be based on observation and recognition of the infant’s needs, cognitive and motor development, interests and desires. However, there is clear evidence that complementary foods should be introduced between 4 and 6 months of age [3,4,5].

The duration of exclusive breastfeeding and the timing of the introduction of complementary foods can influence the weight status of infants, which can persist into childhood [6,7,8]. For the development of prevention programs, interventions, and policies aimed at parent education to address the problem of infant under- and overfeeding, it is necessary to conduct a national survey of a representative sample of infants. The results of national surveys can identify the feeding habits of infants and the factors that influence their feeding habits in their socio-cultural and geographical context [9,10,11,12,13]. According to the available literature, there are a few small-sample, micro-level studies in Croatia that do not provide conclusive insight into infant feeding habits, breastfeeding behavior and complementary feeding [14,15,16,17,18,19,20]. To the authors’ knowledge, there are also no national data on factors influencing infant feeding habits and weight in Croatia.

The first national dietary survey in Croatia for infants, the National Food Consumption Survey on Infants and Children, was carried out between 2017 and 2021 [21]. The survey was supported by the European Food Safety Authority (EFSA) as part of the EU Menu Project. Furthermore, the survey was conducted in accordance with the EFSA methodology [22]. The aim of this study was to provide an overview of dietary patterns and feeding behavior of infants in Croatia based on a representative sample from the national survey in order to close knowledge gaps and gain insights for the development of nutritional guidelines and prevention programs in the field of infant nutrition.

## 2. Materials and Methods

### 2.1. Study Population and Settings

This cross-sectional observational study is part of the National Food Consumption Survey on Infants and Children (OC/EFSA/DATA/2016/02 CT3). The study was conducted from January 2017 to July 2021 according to the Guidance on European Union (EU) Menu Methodology proposed by EFSA [22]. Details of the study design, population and protocol have been described elsewhere [21]. The study was conducted in accordance with the Declaration of Helsinki, and the protocols were approved by the Ethics Committee of the Institute for Medical Research and Occupational Health (No. 100-21/18-9) and by the Croatian Personal Data Protection Agency (No. 567-02/12-18-05). Participation in the study was voluntary. The parents of the infants received and read an information letter and gave their written consent to participate in the study. Participants were free to withdraw their consent at any time during or after data collection.

A stratified random sample at the individual level (sex, age, region) from the databases of the Ministry of Internal Affairs was initially used to draw the population sample. The exclusion criteria were infants who were hospitalized or who lived in different types of shared households (e.g., orphanages). Overall, the study population of The National Food Consumption Survey on Infants and Children comprises a representative sample of 1820 infants and children from Croatia (response rate 22.8%), including 322 (17.7% of the study population) infants aged 3 months to strictly under 12 months, who represent the focus population of the present study.

### 2.2. General Questionnaire

The general questionnaire was completed by the infants’ parents during the face-to-face interview. The interviews were conducted in person or via video call due to the restrictions imposed by the COVID-19 pandemic. As data on sex, age and region were already available in advance, the questionnaire included questions on socio-demographic data (country of birth, nationality, education level and employment status, household size, composition and monthly income), infant birth weight and height, as well as information on breastfeeding (duration of exclusive breastfeeding and breastfeeding habits at the time of the study), infant formula use and complementary feeding (timing of introduction and type of food).

### 2.3. Anthropometry

The anthropometric measurements, body length and body weight were provided by the parents. The measurements were taken during a routine check-up by the pediatrician, either two weeks before or two weeks after the interview with the research team member. Body mass index (kg/m^2^) was calculated from the body length and body weight data. The WHO cut-off values for sex- and age-standardized Z-scores for length, weight and body mass index were used to assess the growth and weight status of the infants [23,24].

### 2.4. Dietary Assessment and Data Processing

Data on the consumption of food, beverages and food supplements were collected on two non-consecutive days using dietary records. All parents/guardians and carers (e.g., grandparents, kindergarten teachers) were instructed on how to keep the dietary records. Dietary records were collected online using NutriCro 2.0 software by parents/guardians or by interviewers if parents/guardians were unable to complete dietary records online. In addition to consumption data, information on preparation methods, brand names and packaging formats was also recorded using the NutriCro 2.0 software. Portion sizes were determined using a validated picture book [25], predefined household measures or standard portion packs, and were all available in the NutriCro 2.0 software or in printed form. The frequency of consumption was recorded for infants who were breastfed at the time of the study. Breast milk intake was quantified according to Paul et al. (1988) for infants up to 10 months, and quadratic interpolation was used for infants over 10 months and up to 12 months [26]. After the day of data collection, the research team reviewed the data entered into the NutriCro 2.0 software. If the researchers found errors, the parents/guardians were contacted to revise the dietary records.

The NutriCro 2.0 software contains the National Food Composition Database [27], supplemented by the nutritional labelling of food products and the Danish Food Database [28] for the nutritional composition of some foods not included in the Croatian Food Composition Database. In the assessment of energy and macronutrients, the intake of food supplements was not taken into account. The estimation of daily energy and macronutrient intake was therefore performed automatically by the software. The method of observed individual means was used to represent daily energy and macronutrient intake [29]. To estimate the relative contribution of each food category to daily energy and macronutrient intake, all foods and beverages were divided into 14 food categories (Supplementary Table S1).

### 2.5. Statistical Analysis

The descriptive analyses are presented separately for boys and girls and for the total sample. All continuous variables are presented as mean and standard deviation, while categorical variables are presented as frequencies or percentages. The Shapiro–Wilk test was used to assess the normality of the distribution of the continuous variables. Initially, there were no missing data in the data set. However, several outliers were identified and replaced by mean values. Imputation of the mean was applied to preserve the overall central tendency of the variables and minimize data loss.

Differences in anthropometric measurements, energy and macronutrient intake, as well as differences between boys and girls in the contribution of food categories to total energy intake, were tested using the *t*-test or the Mann–Whitney U test, depending on the distribution of the variables. In addition, the dependence between socio-demographic characteristics and breastfeeding and complementary feeding habits was tested using the Chi-square test or Fisher’s exact test.

Multiple linear regression analyses with interaction effects were performed to examine the association between dietary patterns (daily energy and daily energy from macronutrients), eating behavior and sociodemographic parameters. Each model used one of the following continuous dependent variables: total daily energy intake or daily energy intake from protein, carbohydrate or fat. Independent variables included: sex (categorical; male as reference), age at month (categorical; <6 months as reference), region (categorical; Dalmatia as reference), household income (categorical; lowest income group as reference), parental education (categorical; lowest education level as reference), breastfeeding (categorical; ‘no’ as reference) and infant formula use (categorical; ‘no’ as reference). Interaction terms were included in two models: (1) age x breastfeeding and (2) age x formula feeding. Although infant age is a quantitative variable, it was categorized into three groups for the regression analysis: (1) <6 months, (2) 6–8 months, and (3) ≥9 months.

A *p*-value of 0.05 was considered significant in all analyses. All statistical analyses and graphical data visualizations were performed with R (version 4.4.2; R Core Team, Vienna, Austria) and IBM SPSS Statistics (version 23.0; IBM Corp., Armonk, NY, USA). The online tool wordart.com was used to create a word cloud depicting the most frequently mentioned first, second and third complementary foods.

## 3. Results

A total of 322 infants (54.0% boys) with an average age of 7.99 ± 2.42 months participated in the National Food Consumption Survey on Infants and Children. On average, the infants had adequate weight status, as shown in Table 1, along with sociodemographic characteristics.

**Figure 1:**
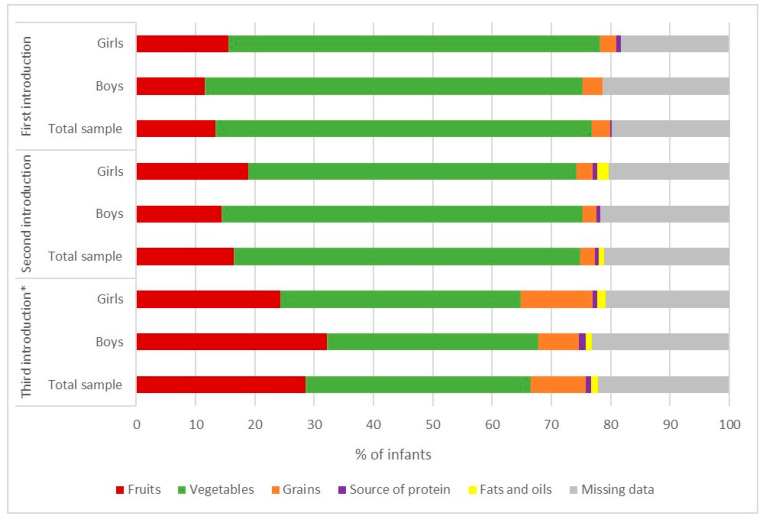
Distribution of infants (n = 322) from the National Food Consumption Survey on Infants and Children who were introduced to different food categories during complementary feeding. * Differences between sexes were tested using the Chi-square test or Fisher’s exact test.

**Figure 2:**
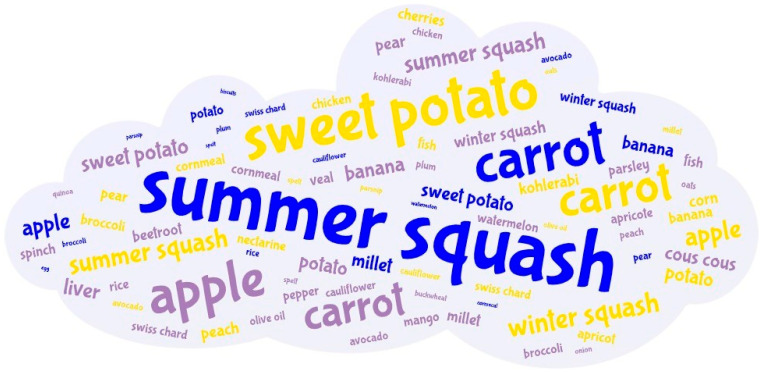
Word cloud of foods that parents include as the first, second and third foods in their infants’ diet (n = 322) from the National Food Consumption Survey on Infants and Children. Foods written in blue are introduced first, yellow second and purple third. A larger font size for the food represents a greater proportion of infants eating this food.

Approximately 30% of the infants were breastfed at the time of the study (Table 2), including more boys than girls (32.8% vs. 31.1%, *p* = 0.023). The infants were exclusively breastfed for an average of 3.85 ± 2.46 months, while the infants were breastfed until approximately 5.26 ± 4.23 months. Half of the study population used infant formula, either in combination with breastfeeding or as exclusively formula feeding. Regarding complementary feeding (Table 2), infants started complementary feeding at 5.37 ± 0.82 months of age, and most infants (73.6%) were introduced to it during the study period. In both sexes, parents mostly started complementary feeding with food items from the vegetable food group (Figure 1). The second food item that parents most frequently included in their children’s diet also cam from the vegetable group. however, the third food item most frequently consumed by the girls came from the vegetable food group (*p* = 0.044), while for the boys it came equally from the fruit and vegetable group. As can be seen in Figure 2, summer squash (44.6%) was most frequently introduced as the first food, followed by sweet potatoes (23.9%) as the second food and apples (17.8%) as the third food.

**Table 2:** Breastfeeding and complementary feeding habits of infants from the National Food Consumption Survey on Infants and Children ^1^.

Characteristics	Total Sample (n = 322)	Boys (n = 174)	Girls (n = 148)	*p* Values *
Breastfeeding habits:				
Breastfeeding at the time of the study, *n* (%)				
No	103 (32.0)	57 (32.8)	46 (31.1)	0.811
Yes	216 (67.1)	116 (67.1)	100 (67.6)	
Missing data	3 (0.9)	1 (0.6)	2 (1.4)	
Exclusively breastfeeding (m.)	3.85 ± 2.46	3.91 ± 2.42	3.78 ± 2.51	0.797
Duration of breastfeeding (m.)	5.26 ± 4.23	5.82 ± 4.25	4.47 ± 2.51	0.069
Complementary feeding:				
Infant formulas, *n* (%)				
No	152 (47.2)	79 (45.4)	73 (49.3)	0.440
Yes	167 (51.9)	94 (54.0)	73 (49.3)	
Missing data	3 (0.9)	1 (0.6)	2 (1.4)	
Start of complementary feeding (m.)	5.37 ± 0.82	5.33 ± 0.80	5.41 ± 0.84	0.261

^1^ All continuous variables are presented as mean and standard deviation; categorical variables as frequencies and percentages. * Differences between the sexes were analyzed using the independent *t*-test or the Mann–Whitney U test, depending on the distribution of the continuous variables, and for categorical variables using the Chi-square test or Fisher’s exact test (*p* < 0.05).

Infants had on average 886 ± 219 kcal of daily energy intake (Table 3), most of which came from carbohydrates, fats and proteins, respectively. No differences in energy and macronutrient intake were observed between the sexes.

**Figure 3:**
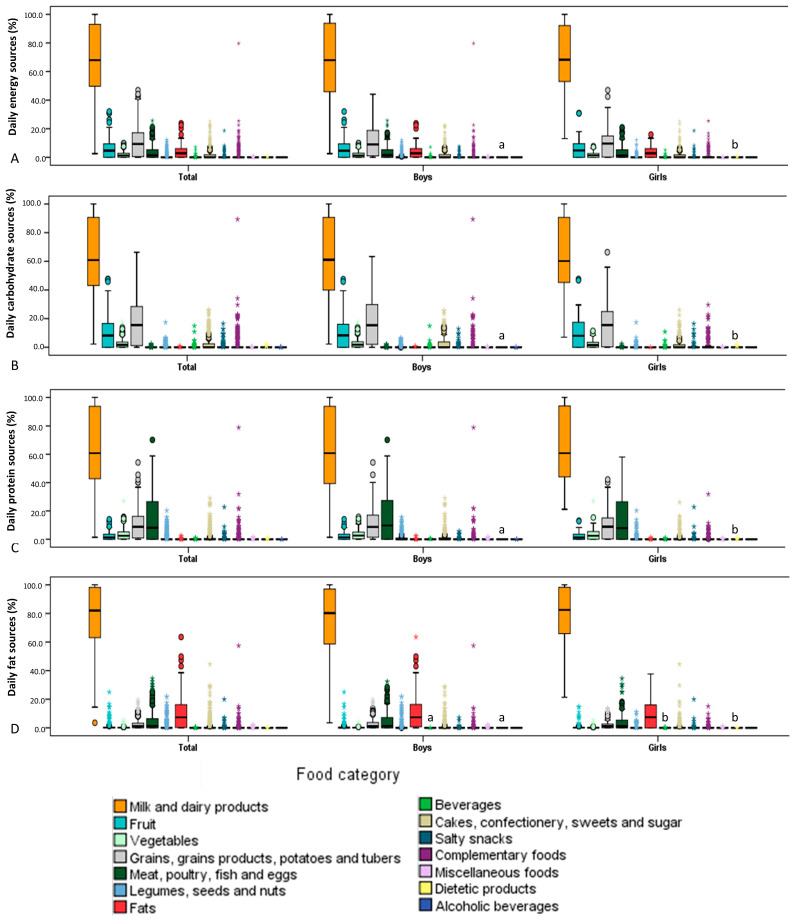
Relative contribution of each food category to the daily energy and macronutrient intake of infants from the National Food Consumption Survey on Infants and Children: (**A**) daily energy intake; (**B**) daily carbohydrate intake; (**C**) daily protein intake; (**D**) daily fat intake. Bars with different letters indicate statistically significant differences between the sexes (Mann-Whitney U test; *p* < 0.05). * Values that are more than 3.0 of interquartile range or above the third quartile.

The food categories that contribute to energy and macronutrient intake are shown in Figure 3. In the total sample of infants, milk and dairy products contributed the most to daily energy intake (68.5%), daily carbohydrate intake (63.7%), daily protein intake (64.9%) and daily fat intake (77.9%). Differences between boys and girls were only found in the contribution of dietetic products to daily energy and macronutrient intake. However, the contribution of dietetic products (supplements and enteral supplements; Supplementary Table S1) to daily energy and macronutrient intake was low. In addition, boys did not consume dietetic products, and only four girls (2.7% of girls in the study population) did.

The final analyses include multivariable regression modeling with interaction effects. The results of the linear regression analysis for each independent variable with total daily energy and daily energy intake from carbohydrates, protein and fats are shown in Supplementary Table S2. Of all the interaction effects examined, two were found to be significant and are shown in detail in Table 4 and Table 5. Specifically, Table 4 shows the results of a multivariable regression model examining the interaction between infant age and breastfeeding in relation to total daily energy intake and daily energy intake from macronutrients. The overall model for total energy intake was statistically significant (F(5, 313) = 13.1, *p* < 0.001) and explained approximately 17.3% of the variance in energy intake. The estimated mean energy intake for non-breastfed infants aged < 6 months was 620.7 kcal/day (95% CI: 534.5 to 706.9, *p* < 0.001), while breastfed infants consumed on average more than 380.8 kcal/day (95% CI: 280.9 to 480.8; *p* < 0.001). However, significant negative interaction effects were observed for the age groups. In the age group of 6 to 8 months and ≥9-month infants, breastfeeding still increased energy intake, but the interaction term indicated that the effect was significantly attenuated compared to infants < 6 months. In terms of daily energy intake from macronutrients, the overall models were significant for carbohydrates (F(5, 313) = 9.61; *p* < 0.001; R2 = 0.1331; β-coefficient: 289.3 kcal/day; 95% CI: 246.3 to 332.3), for proteins (F(5, 313) = 27.3; *p* < 0.001; R2 = 0.3036; β-coefficient: 53.1 kcal/day; 95% CI: 41.4 to 64.7) and for fats ((F(5, 313) = 39.0; *p* < 0.001; R2 = 0.3839; β-coefficient: 274.6 kcal/day; 95% CI: 230.3 to 319.0). Similar attenuating patterns were observed for daily energy intake from macronutrients as for total daily energy intake. These results suggest that the positive association between breastfeeding and energy intake is strongest in early childhood and decreases as the infant gets older.

Table 5 shows the results of a multivariable regression model examining the interaction between infant age and use of infant formula in relation to daily energy intake and intake of daily energy from macronutrients. The overall model for daily energy intake was statistically significant (F(5, 313) = 7.32; *p* < 0.001) and explained approximately 10.47% of the variance in energy intake. In this case, infants < 6 months of age who were not formula-fed consumed 1033.5 kcal/day (95% CI: 970.8 to 1096.2; *p* < 0.001), but formula feeding was associated with a significant decrease in energy intake by an average of 272.2 kcal/day. However, the results suggest that although formula feeding reduces energy intake, the effects diminish with age, leading to increased energy intake in infants aged 6–9 months and >9 months. The overall models for daily energy intake from macronutrients were also significant, with similar age patterns for carbohydrates (F(5, 313) = 8.11; *p* < 0.001; R2 = 0.1147, β-coefficient: 424.4 kcal/day; 95% CI: 393.9 to 454.7), proteins (F(5, 313) = 27.28; *p* < 0.001; R2 = 0.3035; β-coefficient: 76.0 kcal/day; 95% CI: 67.9 to 84.2) and fats (F(5, 313) = 23.7; *p* < 0.001; R2 = 0.2748; β-coefficient: 532 kcal/day; 95% CI: 498.7 to 565.9).

## 4. Discussion

Based on the National Food Consumption Survey on Infants and Children [21], this study provides information on the dietary patterns of infants in Croatia. It is the first study in Croatia that provides insight into infant feeding behavior in a representative sample and fills the knowledge gaps within EU countries, as the study was conducted in accordance with the EU Menu methodology [22]. Furthermore, the results are noteworthy because they provide information on infant feeding habits, a topic that is often overlooked in the literature, as most European studies focus on children aged 1 year and older [30,31,32,33,34,35].

In this population group, the main focus is on breastfeeding and the duration of exclusive breastfeeding [2,3]. The WHO recommends exclusively breastfeeding of infants until the age of 6 months and the continuation of breastfeeding until the age of 2 years, together with the introduction of complementary foods [2]. In the present study, the parents of 61.2% of the infants stated that their infants were exclusively breastfed until an average age of 3.85 ± 2.46 months. About 29.5% of infants were exclusively breastfed at 6 or 7 months of age, while no parent reported that their infants were exclusively breastfed after 7 months of age. These results correspond to some extent with the results of the systematic examinations from 2019, which found that the percentage of exclusively breastfed Croatian infants decreases with age. The percentage of exclusively breastfed infants up to the age of 2 months was 16.6%, while 13.8% and 6.7% were exclusively breastfed at the ages of 3 to 5 months and 6 and 11 months, respectively. None of the infants were reported to be exclusively breastfed after the age of 12 months [14]. The question of exclusive breastfeeding arises in the maternity hospital itself. The study by Miloš et al. (2019) found that between 2005 and 2016, the proportion of mothers exclusively breastfeeding their newborns in maternity wards decreased from 87% to 78.8% and, accordingly, the trend towards combined feeding with breastfeeding and formula increased from 10.3% to 16.1% [15]. The need to increase breastfeeding rates, especially exclusive breastfeeding, in Croatia was already expressed in the first Croatian Nutrition Policy of 1999 [36]. In addition, activities to promote breastfeeding can be found in various health strategies and action plans [37,38,39,40,41]. Until 2020, the Programme for the Protection and Promotion of Breastfeeding was in force, and now the proposal for a new Programme is being prepared [42]. The World Breastfeeding Trends Initiative recently estimated that Croatia is one of the 5 countries within the EU that have a well-implemented Global Strategy for Infant and Young Child Feeding [43]. According to the literature, breastfeeding habits are related to the weight status of infants and consequently, children [6,7,8]. In the present study, the majority of infants (76.4%) had an adequate weight status, while 17.4% of them were overweight and obese. However, a more recent study on a representative sample of children (6 to <10 years old) from Croatia shows a higher rate of overweight (17.6%) and obesity (10.1%) in children [44]. In addition, results from a study of second and third-grade children from Croatia show even higher rates of overweight and obesity, suggesting that children who have been breastfed for less than 6 months are at higher risk for overweight or obesity [45]. Future longitudinal studies on this topic are warranted. They should focus on observing infants over a longer period of time to determine the influence of breastfeeding, but also other feeding habits such as type and amount of complementary foods, on the weight status of infants in Croatia. In the meantime, public health campaigns and programs should continue to promote breastfeeding and the introduction of solid complementary foods at 4 to 6 months of age to prevent overweight and obesity in infants. The results of the European study suggest that infants who are not breastfed or breastfed for shorter periods and infants who are introduced to complementary foods from the age of 7 months have a higher risk of overweight and obesity [46]. This is important because 17.4% of the infants in this study are overweight or obese and, according to the literature, are more likely to remain overweight or obese at preschool age [46].

The infants in the present study consumed an average of 886 ± 219 kcal per day. Compliance with EFSA recommendations for energy and macronutrient intake in infants was not observed as the sample included infants aged 3 months and older [47]. However, in the present study, 75.5% of infants were 6 months or older, and of these, only 25 infants (10.2%) had energy intakes of 90–110% of recommendations, while 86.3% of infants had energy intakes above recommendations. Carbohydrates contributed the most to energy intake (47.0% of total daily energy), followed by fat and protein. In the subgroup of infants over 7 months of age (62.7% of the total infant sample), fat intake ranged from 20.3% to 53.0% of daily energy intake. Only 15 infants from the subgroup had an energy intake from fat of about 40% according to EFSA recommendations, while almost half of the infants exceeded the recommendations [47]. The higher energy intake in infants from 6 months of age has been observed in several European countries, with the same trend in the contribution of macronutrients to daily energy intake [31,32,48,49]. This result suggests a public health intervention to inform the appropriate use of additional fat in infants’ diets and what other sources of fat are present in their diets. Particular attention should be paid to protein intake in infants, especially as previous studies have shown that excessive intake of total protein and animal protein can affect weight status [50,51,52,53]. In the present study, infants had a protein intake of 2.6 ± 0.9 g/kg body weight, and when looking specifically at infants aged 7 months and older, 99% of them were above the EFSA recommendations [54]. This result is in line with previous studies in European countries [31,32,48,55]. This finding points to the need to monitor protein intake in this population and use it for public health strategies to prevent childhood obesity. It is necessary to educate parents about the protein requirements of infants, which foods are sources of protein, and how high the protein content is, especially in infant formula. Parents should also be educated about the effects of higher protein intake on weight status in infancy and later in childhood.

The results of the present study show that the milk and dairy products category contributes 60–80% to energy and macronutrient intake. These results are not surprising since 67.1% of the infants were still breastfed at the time of the study and human milk belongs to the milk and dairy products category. When the diet was analyzed, it was found that the milk subcategory, which includes human and animal milk consumption (only 18.3% of infants consumed animal milk), was the dominant source, accounting for 40–50% of energy and macronutrient intake. A further 20–30% of the energy and macronutrients in this food category came from the infant formula subcategory. The multivariable regression model showed that infant energy and macronutrient intakes differed between breastfed and formula-fed infants. However, these effects vary depending on the age of the infants. Accordingly, the results suggest that breastfeeding has a greater benefit on energy intake in early infancy, while the use of formula is increasing with age. In addition, the models for macronutrient intake showed significant results in both analyses, with similar age-related interactions as for energy intake. The contribution of the other food categories to daily energy and macronutrient intake depended on the age of the child and the complementary food. At the time the study was conducted, the majority of infants (>70%) had already started complementary foods. Parents started complementary feeding when the infants were on average 5.4 ± 0.8 months old, which is in line with recommendations [2,4]. Although the foods for starting complementary feeding are culturally and traditionally predetermined, in most European countries, it is taught that vegetables are the first group of foods for starting complementary feeding, followed by fruits and cereals [56]. In general, parents in the present study introduced vegetables first, followed by fruits and cereals. In most cases, the first foods introduced were different types of squashes, especially summer squash, with sweet potatoes as the second food and apples as the third. This result confirms earlier findings from a smaller study conducted in northwestern Croatia [19]. The available literature has already established that sweet-tasting foods predominate in baby puree, regardless of whether they are homemade or commercial products [56,57,58]. One of the possible reasons for the choice of sweet-tasting foods is that infants have an innate preference for sweetness and accept these foods more readily [13,59]. However, a longitudinal study is needed to determine the reasons for the choice of certain foods during complementary feeding among parents in Croatia and their effects on infants’ later eating habits. In addition, grains, grain products, potatoes and tubers were the second food category that provided an average of 11.0% of energy in both sexes, followed by the fruit category (6.0%). These two food categories were the main source of carbohydrates after the milk and dairy products category. Apart from milk and dairy products, protein intake is largely provided by the consumption of meat, poultry, fish and eggs (14.4%) and grains, grain products, potatoes and tubers (10.4%) groups. In addition, 5.8% of protein comes from the poultry sub-category, 3.6% from the fish and seafood sub-category and only 0.1% from meat products. The consumption of fats contributes to the fats category (10.3%), especially vegetable oils (9.4%), and to the meat, poultry, fish and eggs sub-category (4.4%). Similar to our population, milk (excluding dairy products) is the main source of energy and macronutrients in Belgian infants (6 months to 12 months old) [31]. However, the food category cake and sweets was one of the three main sources of daily energy, carbohydrate and fat intake [31], which is not the case in the present study. In fact, in the present study, the food categories cakes, confectionery, sweeteners and sugar contributed to only 2.1% of daily energy intake and savory snacks to 0.3% of daily energy intake.

One of the greatest strengths of this study is that it includes a representative sample of infants, stratified by gender and region [21] which allows for generalization of conclusions regarding the factors that may influence infants’ eating habits and provides details on the regional diversity of eating habits in relation to the cultural and geographical context of regions in Croatia [60]. However, due to the cross-sectional nature of the study, the causal effect cannot be estimated. All protocols were conducted according to the Guidance on EU Menu methodology proposed by EFSA [22], which minimizes methodological bias. Regarding the methodology, it should be noted that breastfeeding was recorded as the frequency of consumption, and human milk volumes were quantified according to Paul et al. (1988) [26,61], which may lead to an overestimation of human milk consumption and its daily energy contribution for infants with a higher frequency of breastfeeding. To represent daily energy and macronutrient intake, the method of observed individual means from two dietary records was used, which refers to short-term dietary intake [29]. Although measuring the actual weight and length of infants is the most accurate method, anthropometric measurements were self-reported by parents based on pediatricians’ measurements due to the COVID-19 pandemic.

## 5. Conclusions

The average daily energy intake of the children was 882 kcal, with carbohydrates as the main contributor to the daily energy intake, followed by fats and proteins. Milk and dairy products are the main sources of daily energy and macronutrient intake, which highlights their role in infant nutrition. The results emphasize the advantage of breastfeeding in promoting energy and macronutrient intake in early infancy, while the influence of infant formula becomes more favorable as infants grow older. Age-related interactions between energy and macronutrient intake suggest that nutritional interventions should take into account the age-specific needs of infants. Furthermore, the majority of infants had an adequate weight status, while 17.4% of them were overweight or obese. To investigate the long-term effect of feeding habits on the weight status of infants in Croatia, a longitudinal study is warranted. The results of the present study can contribute to the development of prevention programs, strategies and policies that address children’s eating habits and aim for their optimal growth and development.

## Figures and Tables

**Table 1:** Characteristics of infants in the study population from the National Food Consumption Survey on Infants and Children ^1^.

Characteristics	Total Sample	Boys	Girls	*p* Values *
*n* (%)	322 (100.0)	174 (54.0)	148 (46.0)	-
Age (m.)	7.99 ± 2.42	8.11 ± 2.46	7.84 ± 2.38	0.286
Anthropometric characteristics:				
Weight (kg)	8.47 ± 1.45	8.85 ± 1.45	8.03 ± 1.31	<0.001
Length (cm)	70.96 ± 5.24	71.95 ± 5.52	69.79 ± 4.65	<0.001
Body mass index (kg/m^2^)	16.76 ± 1.78	17.06 ± 1.82	16.41 ± 1.67	0.002
z-score weight	0.22 ± 0.97	0.26 ± 1.01	0.16 ± 0.91	0.375
z-score length	0.67 ± 1.49	0.67 ± 1.65	0.67 ± 1.28	0.982
z-score body mass index	−0.21 ± 1.26	−0.15 ± 1.33	−0.27 ± 1.18	0.473
Sociodemographic characteristics:				
Distribution per geographical region, *n* (%)				
Dalmatia	48 (14.9)	27 (15.5)	21 (14.2)	0.707
Istria, Primorje and Gorski Kotar	34 (10.6)	16 (9.2)	18 (12.2)	
Lika and Banovina	19 (5.9)	8 (4.6)	11 (7.4)	
Northern Croatia	31 (9.6)	19 (10.9)	12 (8.1)	
Slavonia	77 (23.9)	40 (23.0)	37 (25.0)	
Zagreb	113 (35.1)	64 (36.8)	49 (33.1)	
Parents’ level of formal education, *n* (%)				
Less than primary education	0 (0.0)	0 (0.0)	0 (0.0)	0.742
Lower primary education	1 (0.3)	0 (0.0)	1 (0.7)	
Upper primary education	5 (1.6)	2 (1.1)	3 (2.0)	
Secondary education	66 (20.5)	33 (19.0)	33 (22.3)	
Graduate or equivalent level	243 (75.5)	135 (77.6)	108 (73.0)	
Doctoral or equivalent level	7 (2.2)	4 (2.3)	3 (2.0)	
Employment status, *n* (%)				
Sick or maternity leave	9 (2.8)	3 (1.7)	6 (4.1)	0.093
Housewife	2 (0.6)	0 (0.0)	2 (1.4)	
Pupil, student or additional training	3 (0.9)	0 (0.0)	3 (2.0)	
Unemployed	45 (14.0)	24 (13.8)	21 (14.2)	
Employed, full-time	263 (81.7)	147 (84.5)	116 (78.4)	
Household income ^2^, *n* (%)				
<378 €	2 (0.6)	1 (0.6)	1 (0.7)	0.256
378–757 €	13 (4.0)	3 (1.7)	10 (6.7)	
758–1141 €	44 (13.7)	25 (14.4)	19 (12.8)	
1142–1513 €	58 (18.0)	32 (18.4)	26 (17.6)	
>1513 €	180 (55.9)	97 (55.7)	83 (56.1)	
Not reported	25 (7.8)	16 (9.2)	9 (6.1)	

^1^ All continuous variables are presented as mean and standard deviation, categorical variables as frequencies and percentages. ^2^ At the time of the study, finances were expressed in Croatian kuna (HRK); the average exchange rate was 1 HRK = 7.53450 EUR. * Differences between the sexes were analyzed using the independent *t*-test or the Mann–Whitney U test, depending on the distribution of the continuous variables, and for categorical variables using the Chi-square test or Fisher’s exact test (*p* < 0.05).

**Table 3:** Average daily energy and macronutrient intake in infants from the National Food Consumption Survey on Infants and Children.

Nutrient	Mean ± SD	P 5	P 25	P 50	P 75	P 95	*p* Values *
Energy (kcal)							
Total sample (n = 332)	886 ± 219	568	629	727	857	1 034	
Boys (n = 174)	891 ± 208	574	641	735	872	1 049	0.421
Girls (n = 148)	880 ± 232	566	609	712	840	1 030	
Carbohydrates (g)							
Total sample (n = 332)	103.6 ± 26.7	64.8	71.0	82.9	101.6	119.6	
Boys (n = 174)	104.7 ± 27.4	63.4	70.0	84.2	103.4	123.9	0.349
Girls (n = 148)	102.2 ± 25.9	67.3	71.3	81.6	100.2	116.6	
Carbohydrates (% kJ)							
Total sample (n = 332)	47.0 ± 6.4	40.5	40.6	41.5	46.0	51.1	
Boys (n = 174)	47.1 ± 6.8	40.4	40.6	41.3	45.9	51.3	0.843
Girls (n = 148)	46.9 ± 5.8	40.6	40.6	41.8	46.2	51.0	
Protein (g)							
Total sample (n = 332)	21.9 ± 8.0	12.0	13.5	16.1	20.2	26.4	
Boys (n = 174)	22.3 ± 7.8	12.7	13.9	16.7	20.8	26.6	0.090
Girls (n = 148)	21.3 ± 8.3	11.2	13.1	15.0	19.6	25.4	
Protein (g/kg BW)							
Total sample (n = 332)	2.6 ± 0.9	1.5	1.7	2.0	2.5	3.0	
Boys (n = 174)	2.5 ± 0.8	1.5	1.7	2.0	2.5	2.9	0.386
Girls (n = 148)	2.7 ± 1.0	1.5	1.6	2.0	2.5	3.2	
Protein (% kJ)							
Total sample (n = 332)	9.9 ± 2.7	7.2	7.3	7.4	9.1	11.7	
Boys (n = 174)	10.1 ± 2.9	7.2	7.3	7.4	9.4	12.3	0.386
Girls (n = 148)	9.6 ± 2.5	7.1	7.3	7.4	8.8	11.2	
Fat (g)							
Total sample (n = 332)	41.6 ± 14.5	22.2	25.3	30.6	38.9	50.4	
Boys (n = 174)	41.3 ± 14.1	22.5	25.1	30.5	39.5	49.5	0.947
Girls (n = 148)	41.8 ± 14.9	21.9	25.3	30.9	38.2	51.5	
Fat (% kJ)							
Total sample (n = 332)	41.9 ± 8.1	27.3	30.4	35.9	42.6	48.9	
Boys (n = 174)	41.5 ± 8.7	24.5	29.1	34.6	42.6	49.8	0.619
Girls (n = 148)	42.3 ± 7.2	29.0	32.2	37.0	42.6	48.2	

* Differences between the sexes were analyzed using the independent *t*-test or the Mann-Whitney U test, depending on the distribution of the continuous variables (*p* < 0.05).

**Table 4:** Multivariable regression models including interaction between age and breastfeeding in infants from the National Food Consumption Survey on Infants and Children.

Variable	Age 6–8 Months	Age ≥ 9 Months	Breastfeeding (Yes)	Interaction (6–8 Months x Breastfeeding)	Interaction (≥9 Months x Breastfeeding)
Energy (kcal/day)					
β coefficient (95% CI)	169.2 (59.6–278.8)	281.4 (178.1–384.7)	380.85 (280.9–480.8	−267.26 (−396.8–−137.8)	394.7 (−517.6–−271.9)
*p*-values	0.002	<0.001	<0.001	<0.001	<0.001
Carbohydrate (kcal/day)					
β coefficient (95% CI)	128.3 (73.6–182.9)	180.6 (129.1–232.2)	123.6 (73.7–173.4)	−132.3 (−196.9–−67.7)	−173.6 (−234.8–−112.3)
*p*-values	<0.001	<0.001	<0.001	<0.001	<0.001
Protein (kcal/day)					
β coefficient (95% CI)	26.5 (11.7–64.7)	59.1 (45.1–73.0)	20.8 (7.3–34.3	−23.9 (−41.4–−6.4)	−28.3 (−44.9–−11.7)
*p*-values	<0.001	<0.001	0.002	0.007	<0.001
Fat (kcal/day)					
β coefficient (95% CI)	6.1 (−50.3–62.5)	27.9 (−25.3–81.1)	239.4 (187.6–290.5)	−110.2 (−176.8–−43.5)	−192.9 (−256.2–−129.7)
*p*-values	0.832	0.302	<0.001	0.001	<0.001

**Table 5:** Multivariable regression models including interaction between age and consumption of infant formula in infants from the National Food Consumption Survey on Infants and Children.

Variable	Age 6–8 Months	Age ≥ 9 Months	Infant Formula (Yes)	Interaction (6–8 Months x Infant Formula)	Interaction (≥9 Months x Infant Formula)
Energy (kcal/day)					
β coefficient (95% CI)	−157.2 (−242.6–−71.7)	−142.9 (−225.3–−60.5)	−272.2 (−363.1–−181.3)	253.9 (132.7–375.2)	277.0 (161.1–392.9)
*p*-values	<0.001	<0.001	<0.001	<0.001	<0.001
Carbohydrate (kcal/day)					
β coefficient (95% CI)	−32.4 (−73.8–8.9)	−6.8 (−46.7–33.1)	−90.7 (−134.7–−46.7)	128.4 (69.6–187.2)	127.8 (71.6–183.9)
*p*-values	0.124	0.739	<0.007	<0.001	<0.001
Protein (kcal/day)					
β coefficient (95% CI)	−2.1 (−13.2–−9.0)	26.6 (15.8–37.2)	−15.8 (−27.6–−3.9)	22.5 (6.8–38.3)	24.3 (9.3–39.4)
*p*-values	0.711	<0.001	0.009	0.005	0.001
Fat (kcal/day)					
β coefficient (95% CI)	−129.5 (−175.4–−83.6)	−177.5 (−221.7–−133.3)	−167.8 (−216.6–−119.1)	101.2 (36.1–166.3)	126.4 (64.2–188.7)
*p*-values	<0.001	<0.001	<0.001	0.002	<0.001

## Data Availability

The data that support the findings of this study are available from the EU Menu team, but restrictions apply to the availability of the raw data, which were used under license for the current study and so are not publicly available. Data are, however, available from the authors upon reasonable request and with permission.

## Supplementary Materials

The following supporting information can be downloaded at: https://www.mdpi.com/article/10.3390/children12091125/s1, Table S1: The list of food categories and their belonging food subcategories; Table S2: The regression analysis for each independent variable with daily energy and daily energy intake from macronutrients in infants from the National Food Consumption Survey on Infants and Children.

## Abbreviations

The following abbreviations are used in this manuscript:

EFSA	European Food Safety Authority
EU	European Union
WHO	World Health Organization
