# Supplementary material for: Dietary Patterns and Feeding Behavior of Infants in Croatia: Findings from the National Food Consumption Survey on Infants and Children
Source: Children (Basel). 2025 Aug 26;12(9):1125. doi: 10.3390/children12091125 (PMC12468696; doi:10.3390/children12091125)
Supplement: Supplementary file 1 [file children-12-01125-s001.zip › Supplementary Table S1.pdf]

Supplementary Table S1. The list of food categories and their belonging food subcategories

Food category	Food subcategory
Milk and dairy products	Human milk<br>Infant formulas<br>Milk<br>Flavoured milk<br>Fermented dairy products<br>Flavoured fermented dairy products<br>Dairy desserts<br>Cheese and cheese spreads
Fruit	Fresh, canned, frozen fruits<br>100% fruit juices<br>Dried fruits and fruit bars (% fruit)
Vegetables	Fresh, canned, frozen vegetables<br>100% vegetables juices<br>Dried vegetables
Grains, grain products, potatoes and tubers	Bread, rolls and tortillas<br>Grains, grits and flour<br>Pasta<br>Breakfast cereals<br>Fresh and frozen potatoes and tubers<br>Fresh and frozen potatoes and tubers products
Meat, poultry, fish and eggs	Fresh and frozen read meat and poultry<br>Fresh and frozen fish and seafood<br>Eggs<br>Meat, poultry and fish products (processed, canned or breaded)
Legumes, seeds and nuts	Fresh, dried, canned and frozen legumes<br>Seeds and seeds products without added salt and sugar<br>Nuts and nuts products without added salt and sugar<br>Milk and meat alternatives
Fats	Plant oils<br>Animal fats
Complementary foods	Ready-to-eat baby porridges and desserts made of fruit and vegetables<br>Read–to-eat baby meals<br>Ready-to-eat cereal and milk porridges
Cakes, confectionery, sweets and sugar	Chocolate and chocolate spreads with additives<br>Cocoa powder<br>Biscuits and dry cakes<br>Cakes and tarts<br>Marmalades, jams and jellies<br>Honey and sweeteners<br>Ice creams based on water and/or milk<br>Sugar<br>Confectionery and chewing gum

	Other
Salty snacks	Grain based salty snacks<br>Potato based salty snacks
Beverages	Water<br>Tea<br>Fruit and vegetable juices<br>Other non-alcoholic beverages
Miscellaneous foods	Salt<br>Condiment with dehydrate vegetables, spices and salt<br>Yeast<br>Dehydrated stocks
Dietetic products	Supplements<br>Enteral supplementation
Beverages	Water<br>Tea<br>Fruit and vegetable juices<br>Other non-alcoholic beverages
Alcoholic beverages	Wine<br>Vinegar
