# Supplementary material for: Dietary Patterns and Feeding Behavior of Infants in Croatia: Findings from the National Food Consumption Survey on Infants and Children
Source: Children (Basel). 2025 Aug 26;12(9):1125. doi: 10.3390/children12091125 (PMC12468696; doi:10.3390/children12091125)
Supplement: Supplementary file 1 [file children-12-01125-s001.zip › Supplementary Table S2.pdf]

Supplementary Table S2. The regression analysis for each independent variable with daily energy and daily energy intake from macronutrients in infants from the National Food Consumption Survey on Infants and Children

Variables	Energy (kcal/day)		Carbohydrates (kcal/day)		Protein (kcal/day)		Fat (kcal/day)	
	β coefficient (SE)	p - Values	β coefficient (SE)	p - Values	β coefficient (SE)	p - Values	β coefficient (SE)	p - Values
**Age:**	**904.1 (24.2)**	**< 0.001**	**381.2 (11.6)**	**< 0.001**	**68.5 (3.0)**	**< 0.001**	**452.5 (13.4)**	**< 0.001**
Age < 6 months	Ref.							
Age 6 – 8 months	-38.1 (32.2)	0.238	30.3 (15.4)	0.049	8.9 (4.0)	0.027	-85.2 (17.8)	< 0.001
Age ≥ 9 months	-13.3 (30.79)	0.668	55.7 (14.7)	< 0.001	38.6 (3.9)	< 0.001	-121.6 (17.1)	< 0.001
**Sex:**	**890.8 (16.63)**	**< 0.001**	**418.9 (8.1)**	**< 0.001**	**89.4 (2.4)**	**< 0.001**	**371.9 (9.9)**	**< 0.001**
Boys	Ref.							
Girls	-10.7 (24.5)	0.664	-10.0 (11.9)	0.403	-4.3 (3.6)	0.238	4.4 (14.6)	0.765
**Region:**	**867.1 (31.7)**	**< 0.001**	**406.9 (15.3)**	**< 0.001**	**81.8 (4.6)**	**< 0.001**	**368.9 (18.9)**	**< 0.001**
Dalmatia	Ref.							
Istria, Primorje and Gorski Kotar	21.1 (49.2)	0.668	-3.5 (23.8)	0.881	10.0 (7.2)	0.162	14.0 (29.3)	0.632
Lika and Banovina	-43.2 (59.45)	0.468	-9.1 (28.7)	0.752	6.7 (8.7)	0.438	-43.9 (35.4)	0.216
Northern Croatia	79.6 (50.5)	0.116	60.2 (24.4)	0.014	18.3 (7.4)	0.013	-1.1 (30.1)	0.970
Slavonia	15.1 (40.3)	0.709	11.8 (19.5)	0.546	3.4 (5.9)	0.561	0.5 (24.0)	0.984
Zagreb	22.6 (37.8)	0.551	-0.8 (18.3)	0.966	4.5 (5.5)	0.413	17.6 (22.5)	0.436
**Household income:**	**865.5 (43.9)**	**< 0.001**	**414.2 (21.3)**	**< 0.001**	**89.7 (6.4)**	**< 0.001**	**349.8 (26.1)**	**< 0.001**
< 378 €	Ref.							
378 – 757 €	-42.9 (74.9)	0.567	-37.3 (36.3)	0.306	-15.8 (10.9)	0.151	14.0 (44.6)	0.754

756 – 1141 €	13.1 (54.9)	0.812	14.7 (26.6)	0.582	-3.2 (8.1)	0.689	3.9 (32.7)	0.906
1 142 – 1 513 €	49.5 (52.5)	0.346	26.3 (25.4)	0.302	1.4 (7.7)	0.858	21.6 (31.2)	0.489
> 1 513 €	17.8 (46.8)	0.704	-10.1 (22.7)	0.657	-2.9 (6.9)	0.674	32.6 (27.9)	0.243
**Parents level of formal education:**	**551.5 (217.4)**	**0.011**	**277.3 (106.9)**	**0.009**	**43.5 (32.2)**	**0.177**	**226.3 (129.3)**	**0.081**
Lower primary education	Ref.							
Upper primary education	383.3 (238.2)	0.109	141.6 (117.1)	0.227	42.4 (35.3)	0.230	195.7 (141.7)	0.168
Secondary education	286.8 (219.1)	0.192	123.0 (107.7)	0.254	41.8 (32.4)	0.198	116.4 (130.0)	0.372
Master's or equivalent level	350.8 (217.9)	0.108	142.0 (107.1)	0.185	45.1 (32.3)	0.163	157.9 (129.6)	0.223
Doctoral or equivalent level	226.1 (232.5)	0.331	113.1 (114.3)	0.323	31.5 (34.4)	0.360	75.2 (138.2)	0.586
**Breastfeeding:**	**807.7 (20.9)**	**< 0.001**	**415.8 (10.5)**	**< 0.001**	**89.3 (3.2)**	**< 0.001**	**289.7 (11.5)**	**< 0.001**
No	Ref.							
Yes	117.5 (25.4)	< 0.001	-1.5 (12.8)	0.904	-2.7 (3.9)	0.491	125.6 (13.9)	< 0.001
**Infant formulas:**	**926.3 (17.5)**	**< 0.001**	**411.1 (8.7)**	**< 0.001**	**85.6 (2.6)**	**< 0.001**	**420.7 (9.9)**	**< 0.001**
No	Ref.							
Yes	-74.5 (24.2)	0.002	7.1 (11.9)	0.553	3.6 (3.6)	0.323	-87.9 (13.8)	< 0.001
